# DNA Walkers for Biosensing Development

**DOI:** 10.1002/advs.202200327

**Published:** 2022-04-22

**Authors:** Lu Song, Ying Zhuge, Xiaolei Zuo, Min Li, Fang Wang

**Affiliations:** ^1^ Department of Cardiology Shanghai General Hospital School of Medicine Shanghai Jiao Tong University Shanghai 200800 China; ^2^ Institute of Molecular Medicine Shanghai Key Laboratory for Nucleic Acid Chemistry and Nanomedicine School of Medicine Shanghai Jiao Tong University Shanghai 200127 China

**Keywords:** biosensors, DNA walkers, driving forces, walking legs

## Abstract

The ability to design nanostructures with arbitrary shapes and controllable motions has made DNA nanomaterials used widely to construct diverse nanomachines with various structures and functions. The DNA nanostructures exhibit excellent properties, including programmability, stability, biocompatibility, and can be modified with different functional groups. Among these nanoscale architectures, DNA walker is one of the most popular nanodevices with ingenious design and flexible function. In the past several years, DNA walkers have made amazing progress ranging from structural design to biological applications including constructing biosensors for the detection of cancer‐associated biomarkers. In this review, the key driving forces of DNA walkers are first summarized. Then, the DNA walkers with different numbers of legs are introduced. Furthermore, the biosensing applications of DNA walkers including the detection‐ of nucleic acids, proteins, ions, and bacteria are summarized. Finally, the new frontiers and opportunities for developing DNA walker‐based biosensors are discussed.

## Graphical abstract

In this review, the key forces of driving DNA walkers are summarized. Besides, a variety of DNA walkers with different numbers of legs are discussed. And DNA walkers‐based biosensors are given examples, including nucleic acids biosensors, proteins biosensors, ions biosensors, and bacteria biosensors. Eventually, the challenges and opportunities of developing DNA walker‐based biosensors are discussed.

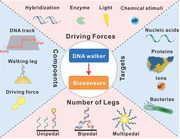

## 1 Introduction

During the past several years, nucleic acids (DNA and RNA) have been identified as promising nanomaterials in the biological field.^[^ ^1^, ^2^, ^3^ ^]^ The DNA strand can specifically hybridize with its complementary strand spontaneously, which makes it hold the excellent molecular recognition ability. As a nanomaterial, DNA exhibits lots of strengths: stability, specificity, programmability, and biocompatibility.^[^ ^4^, ^5^, ^6^ ^]^ Taking these great advantages, DNA nanotechnology has moved from proof‐of‐concept structural constructs to biological applications. For example, DNA sequence programmability allows scientists to design precise nanostructures to fabricate many biosensors with the aid of the strict “Waston‐Crick” pairing principle.^[^ ^7^, ^8^, ^9^ ^]^ DNA‐based biosensors have been applied in biological, medical, and chemical applications. In recent years, dynamic DNA nanostructures have attracted extensive attention and various dynamic DNA nanostructures have been developed, such as DNA tweezers,^[^ ^10^ ^]^ DNA walkers,^[^ ^11^ ^]^ and DNA nanorobots.^[^ ^12^ ^]^ The transformation of dynamic DNA nanostructures is controllable. In detail, when stimulated by molecular triggers (such as ions, nucleic acids, enzymes, and various chemical stimuli), dynamic DNA nanostructures exhibit directional movement or complicated behavior in specific ways.^[^ ^13^, ^14^, ^15^, ^16^ ^]^

**Scheme 1:**
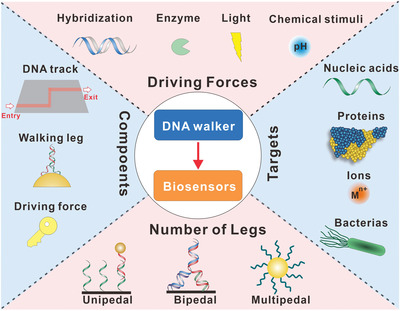
Overview of the construction and biosensing application of DNA walkers.

DNA walkers, as one of the dynamic DNA nanodevices, have recently attracted intense interest in designing and fabricating DNA‐based biosensors.^[^ ^17^, ^18^, ^19^, ^20^ ^]^ Typical DNA walkers are mainly composed of three major elements including the driving forces, the walking strands, and the walking tracks. Once the driving forces are introduced, the initial equilibriums of DNA walkers are broken, and then the chemical energy or light energy is converted into mechanical energy, which drives the DNA walkers moving along the walking track. By consuming fuel molecules, the equilibriums are developed again. Subsequently, the signals are generated. Due to the fact that the equilibriums of DNA walkers can be broken and redeveloped sequentially, the signal output of the DNA walkers can be amplified eventually.^[^ ^21^, ^22^, ^23^ ^]^ Based on the signal amplification capability of DNA walkers, researchers have constructed a variety of DNA walker‐based biosensors for the detection of various analytes, such as nucleic acids,^[^ ^24^ ^]^ ions,^[^ ^25^ ^]^ small molecules, proteins, and circulating tumor cells (CTCs) (**Scheme** **1**).^[^ ^26^, ^27^, ^28^ ^]^ Due to the fact that DNA walkers are a type of dynamic molecular devices, it is suitable to activate the autonomous movement of DNA walkers in living cells. The spontaneous movement of DNA walkers allows to accomplish the real‐time imaging of cells by intracellular operation, which may inspire diverse biological applications including drug delivery, diagnosis, etc.

In this review, we introduced the design principles and driving forces of DNA walkers. Typical driving forces of DNA walkers, including strand displacement reaction, enzymatic reaction, optical and chemical stimulus are summarized. Besides, we discussed different number of legs for DNA walkers. A set of unipedal, bipedal and multipedal DNA walkers were presented. Then we described the sensing applications of the DNA walkers in recent several years, such as sensing various biomarkers and delivering drugs or cargos. For each case, we summarized the characteristics of DNA walkers in biosensing, including the strengths and shortcomings of the systems. Finally, we discussed the main challenge of DNA walkers for analyzing biomarkers, and provided future directions of DNA walker‐based biosensors.

## 2 Driving Force for DNA Walkers

Since the first DNA nanostructures were constructed by Seeman in 1998, the DNA nanotechnology for designing DNA‐based nanomotors has been developed rapidly in recent several decades.^[^ ^29^, ^30^, ^31^, ^32^ ^]^ The programmable assembly of DNA nanostructures has made DNA walkers suitable for moving at molecular level. In biosensing systems, the events of recognizing or binding biomarkers could be converted into walking rate and steps of DNA walkers walking. Furthermore, outstanding performance of DNA walkers in sensitivity and addressability are desirable in biological analysis. Therefore, it is important for researchers to identify energy supplies in the development of the DNA walkers. To drive DNA walkers moving along 1D or 2D or 3D DNA tracks effectively, driving forces are indispensable.^[^ ^33^, ^34^, ^35^ ^]^ At present, the main driving forces of DNA walkers are strand displacement reactions, enzymatic (protein enzyme or DNAzyme) reactions, photoinitiated reactions, and chemical stimulus reactions. The categories of driving force are generally determined by the properties of targets. To achieve better performance, a variety of driving forces are raised. In this section, we mainly discuss DNA walkers driven by the strand displacement reactions, the enzymatic reactions, the photoinitiated reactions, and the chemical stimulus.

### 2.1 Strand Displacement Reactions‐Based DNA Walkers

The conduction of strand displacement reactions is based on the principle of strict complementary base pairing.^[^ ^36^, ^37^ ^]^ Strand displacement reactions‐based methods are employed for driving DNA walkers effectively due to the high controllability and programmability of DNA self‐assembled nanostructures.^[^ ^38^, ^39^ ^]^ DNA strands displacement reactions are accomplished by the competition of hybridization and de‐hybridization, which promotes the kinetic and thermodynamics equilibrium through the hybridization between the walking strand and the substrate strand or the fuel strand.^[^ ^40^ ^]^ The exposed toehold of walking strand is accessible to integrating with downstream reaction.^[^ ^41^ ^]^ Strand displacement reactions play important roles in driving DNA walkers and achieving strand displacement reactions‐dependent behaviors.

**Figure 1:**
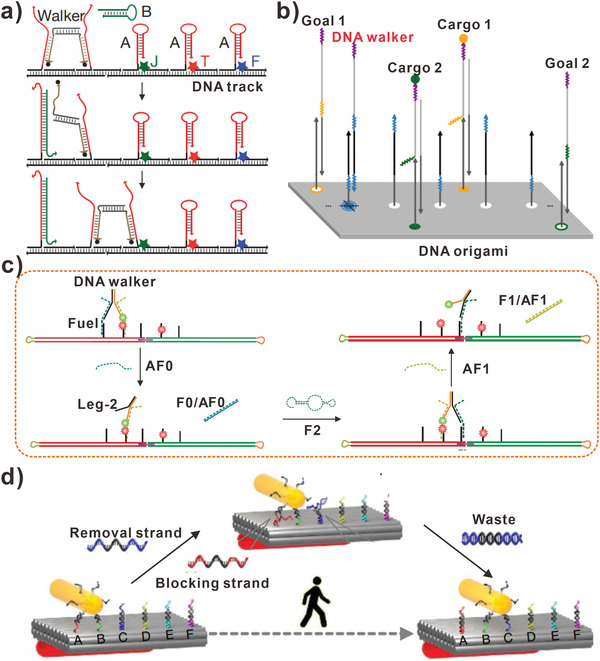
DNA walkers driven by strand displacement reactions. a) DNA walkers moved along a linear DNA track. Reproduced with permission.^[^ ^42^ ^]^ Copyright 2008, Springer Nature. b) Cargo‐sorting DNA walkers moved on a DNA origami. Reproduced with permission.^[^ ^43^ ^]^ Copyright 2017, American Association for the Advancement of Science. c) DNA walkers traveled between two origami tiles. Reproduced with permission.^[^ ^44^ ^]^ Copyright 2014, Wiley‐VCH. d) Plasmonic nanorod‐comprised DNA walkers. Reproduced with permission.^[^ ^45^ ^]^ Copyright 2015, Springer Nature.

In recent years, many researches of simple strand displacement reaction have been developed to design DNA walkers. **Figure** **1**a shows a DNA walker constructed by Pierce and his group in 2008.^[^ ^42^ ^]^ In this research, they used a “reaction graph” abstraction to program different molecular self‐assembly and disassembly pathway. The “reaction graph” was used for specifying complementary relationships between modular domains in the universal DNA hairpin motif. They developed an autonomous enzyme‐free DNA walker that was capable of stochastic moving along a linear DNA track. The DNA track consisted of hairpins namely A at certain intervals along a nicked DNA duplex. In the presence of complementary strand B, the walker was expected to move along the linear DNA track unidirectionally. Based on the arrangement of anchor sites in 1D track, the fuel strand B hybridized with the exposed toehold of the first strand anchored on the DNA track. Then the leg of the bipedal DNA walker was released and reattached another strand A to form the new duplex. The fixed‐release‐fixed cycles could be achieved by mixing the strand B continuously. Furthermore, the DNA walker would be more functional by integrating with a variety of biomarker identification sequences. Almost at the same time, Seeman et al. designed a bipedal DNA walker with coordinated legs.^[^ ^46^ ^]^ The leading leg of DNA walker catalyzed the release of trailing leg.

Consequently, the bipedal DNA walker moved by walking along the DNA track. By controlling toehold interactions kinetically, the DNA walker could take two steps spontaneously from resting‐state 1 (RS‐1) to resting‐state 3 (RS‐3). When only fuel strand F1 was added, the one‐step transition from RS‐1 to RS‐2 occurred. However, no transition took place when F2 was added alone, which demonstrated the control of the walking system. In recent years, DNA origami has been developed for constructing biocompatible platforms. DNA origami can be controllably designed and produced with uniform sizes and shapes, as well as excellent programmability. Due to the fact that various cargos can be placed on DNA origami with desired numbers and patterns, Lulu Qian and colleagues designed a cargo‐sorting DNA walker (Figure 1b).^[^ ^43^ ^]^ To pick up different cargos that were placed on DNA origami at unordered locations and deliver each type of cargos to the desired place, the DNA walker with three modular building blocks was constructed. With the aid of DNA strand displacement reaction, the DNA walker achieved three processes, walking randomly, picking up cargo, and dropping off cargo. On the addressable DNA origami, the DNA walker walked about 300 steps while sorting two types of cargos. Furthermore, when a new building block on a path was added, the DNA walker could find the shortest path and transport cargo molecules efficiently, which performed like macroscopic robots. However, the size of origami is restricted by the length of scaffold that usually contains 7249 nucleotides. To address the size limitation, origami has been joined together by a set of DNA strands. In order to construct DNA walkers that walked more than one origami, Eyal Nir and colleagues designed a bipedal DNA walker that can move from one DNA origami tile to another and then back again (Figure 1c).^[^ ^44^ ^]^ By joining together with a series of single‐strands, two different DNA origami tiles formed a stable DNA track for DNA walker traveling. At the first time, the bipedal DNA walker was attached to one of the two DNA origami tiles, then it moved from one DNA origami tile to another spontaneously. The walking process was conducted by hybridizing and displacing fuel DNA strands. By continuously introducing fuel strands, the bipedal DNA walker was directed to move from one DNA origami to another and then back to the first DNA origami. Eventually, the DNA walker moved 64 nm in the walking process. Based on the research, DNA walkers that can move over micrometer distances will be developed with more DNA origami.

Typically, it is difficult for researchers to visualize the process of DNA walkers moving along precisely defined DNA tracks. But there were more processes of walking along microparticle surfaces need to be understood clearly. In 2015, Zhou et al. reported a DNA walker that comprised a plasmonic nanorod walked on the DNA origami (Figure 1d).^[^ ^45^ ^]^ The DNA walker comprised an anisotropic gold nanorod and discrete DNA strands. In this assay, the walking directions and steps of the DNA walker could be reported at nanometer accuracy with the aid of optical information carried by gold nanorod. The combination of DNA nanostructure and plasmonic nanorod suggested that the construction of artificial synthetic machines was feasible. The artificial DNA walkers showed their structural dynamics in situ using stable and optical approach, which enabled to render profound significance in dual disciplines. Furthermore, the concept of synthetic DNA walkers also outlined an outstanding prospect of developing programmable large‐scale DNA nanomotors that incorporated electrical and biochemical components for information transmitting and cargo transporting. To transport different cargos, Seeman and his work team proposed a nanoscale assembly line that combined three DNA‐based modules: DNA origami tile, DNA walker, and the cassettes containing three independently controlled DNA machines for programmable cargo donating.^[^ ^47^ ^]^ Three different sizes of gold nanoparticles were served as cargos and eight different products were constructed.

### 2.2 Enzymatic Reactions Based‐DNA Walkers

The above‐mentioned DNA walkers were typically driven by externally supplied DNA single‐strands. The hybridization between DNA single‐strands provides chances for changing conformation and walking along DNA tracks of DNA walkers. Meanwhile, as a typical driving force, enzymatic reactions were also introduced to the field of designing DNA walker.^[^ ^52^, ^53^ ^]^ Enzyme‐powered DNA walkers have become popular in recent years.^[^ ^54^ ^]^ The enzymes act on DNA phosphate backbone, and the free energy produced by the cleavage of covalent bonds drives DNA walkers to move.^[^ ^55^ ^]^ Endonuclease,^[^ ^56^ ^]^ exonuclease,^[^ ^57^, ^58^ ^]^ and DNAzyme^[^ ^59^, ^60^, ^61^ ^]^ are classic enzymes used in driving DNA walkers. In this section, we will summarize the DNA walkers driven by enzymatic reactions.

**Figure 2:**
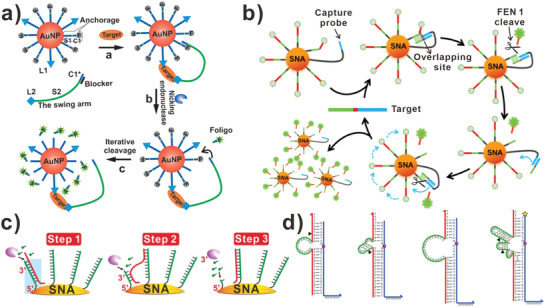
DNA walkers driven by enzymatic reactions. a) Nicking endonuclease‐driven DNA walkers. Reproduced with permission.^[^ ^48^ ^]^ Copyright 2015, Wiley‐VCH. b) Flap endonuclease 1‐driven DNA walkers. Reproduced with permission.^[^ ^49^ ^]^ Copyright 2021, American Chemical Society. c) DNA walkers driven by exonuclease. Reproduced with permission.^[^ ^50^ ^]^ Copyright 2017, Wiley‐VCH. d) DNA walkers driven by different DNAzymes. Reproduced with permission.^[^ ^51^ ^]^ Copyright 2015, American Chemical Society.

In 2011, Andrew et al. introduced a DNA walker that was powered by nicking restriction enzyme.^[^ ^62^ ^]^ Nicking restriction enzyme is a class of enzyme that only cuts one strand of the duplex DNA, and leaves the contact DNA strand for the following reaction. The DNA walker proposed by Andrew was a single‐strand DNA, which was complementary to the stator. Meanwhile, the stator duplex contained the recognition site of nicking restriction enzyme. When the backbone of stator was hydrolyzed by nicking restriction enzyme, the DNA walker was driven by the released energy and moved from the cut stator to the next intact stator. Finally, the DNA walker walked along the 100‐nm‐long DNA track on a 2D origami. With the aid of real‐time atomic force microscopy, the individual steps and mechanistic details of a single DNA walker were observed. The DNA track comprised 16 steps in the DNA origami and moved from one end of the track to the other end at an average speed. Besides walking along 1D or 2D track, there were many researches in developing DNA walkers moved along 3D track. In 2015, Zhang et al. proposed a DNA walker that could be hybridized to the DNA track on the surface of gold nanoparticles (AuNPs) in the presence of proteins, small molecules, and nucleic acids (**Figure** **2**a).^[^ ^48^ ^]^ They constructed 3D DNA tracks on AuNPs, which functionalized with a series of DNA single‐strands and affinity ligands. In this assay, the DNA walker was linked to the affinity ligand. When bound to the target molecule, the DNA walker was brought to AuNPs and the movement around the AuNP surface was initiated spontaneously at the aid of nicking endonuclease‐based reactions. In 2016, Li et al. designed a DNA walker immobilized on the surface of AuNPs with affinity ligand, which tremendously improved the velocity of amplifying signals.^[^ ^63^ ^]^ Recently, Fang et al. proposed a 3D DNA walker applied in biosensing.^[^ ^35^ ^]^ The movement of DNA walker was promoted by the nicking endonuclease‐based reaction. Furthermore, the DNA walker could be used in detecting target DNA with high specificity in the detection range of 10 pM–5 nM. However, it is challenging for DNA walkers to distinguish DNA targets with single‐base differences. Cheng et al. developed a DNA walker powered by a flap endonuclease 1 (FEN 1) to achieve mutant DNA biosensing (Figure 2b).^[^ ^49^ ^]^ The target DNA was conducted as DNA walker to hybridize with the DNA track on the surface of AuNP. Then the FEN 1 could cleave the DNA track strands that formed a three‐base overlapping structure after hybridized with target DNA. Based on the high specificity of FEN 1, the DNA walker system could be used to discriminate one‐base mutant DNA target from wild‐type DNA and 0.1% mutation could be sensed, indicating the potential for liquid biopsy.

Unlike endonuclease, exonuclease cleaves the phosphodiester bond from the end of DNA chain. Due to that exonuclease does not require the recognition sties, the design of DNA walker system can be more flexible. Fan and his team reported a stochastic DNA walker powered by exonuclease III (Exo III) that moved on the surface of spherical nucleic acid (SNA) (Figure 2c).^[^ ^50^ ^]^ The Exo III digested the hybridized DNA tracks, leaving the DNA walker moved unidirectionally. In this assay, the process of walking was monitored by total internal reflection fluorescence microscopy at the single‐particle resolution in real‐time. Given that nuclease activity was related to the DNA density and the SNA conformation, the performance of DNA walker was dependent upon the morphology of the system. Finally, the Exo III powered DNA walker exhibited high sensitivity of 10 fM in detecting target DNA. Meanwhile, He et al. developed Exo III‐assisted target recycling amplification (ERA) strategy to construct DNA walker biosensor for the detection of carcinoembryonic antigen (CEA).^[^ ^64^ ^]^ The detection limit of CEA can be as low as 1.2 pg mL^−1^. And satisfactory results can be obtained when analyzing human serum sample, showing great potential in clinical diagnosis.

DNAzyme, also known as DNA enzyme, is a type of single‐stranded DNA with catalytic capability. DNAzyme is selected in vitro and hundreds of DNAzymes have been isolated in recent twenty years. With the aid of metal ions or small molecules, DNAzyme can drive the spontaneous motion of DNA walker without fuel molecules. Hyun Choi and colleagues developed a DNA walker that transported CdS nanoparticles along single‐walled carbon nanotubes (SWCNTs) in 2013.^[^ ^65^ ^]^ The movement of DNA walker was based on RNA‐cleaving DNA enzymatic reaction. The chemical energy originated from cleaved RNA molecules fueled DNA walker moving along the 1D track spontaneously. Given that the activity of DNAzyme depended on the presence of Mg^2+^, the walking process was controllable, which allowed to direct “go” and “stop” actions remotely. In this assay, the DNA walker moved near 3 µm at an average speed of ∼1 nm min^−1^. Subsequently, Hyun Choi et al. provided design principles of DNAzyme‐based DNA walker (Figure 2d).^[^ ^51^ ^]^ They performed a theoretical model to demonstrate that several key parameters govern the kinetics of DNAzyme‐based walker. The main parameters included DNAzyme structure and core type, recognition arm lengths, metal cation species, and concentration. For instance, the efficiency of cleavage reaction was increased twofold when the DNAzyme catalytic cores opened under the irradiation of UV. After optimizing these parameters, the DNA walker powered by DNAzyme moved 5 µm at an average speed of ∼1 nm s^−1^.

### 2.3 Other Stimulus‐Based DNA Walkers

**Figure 3:**
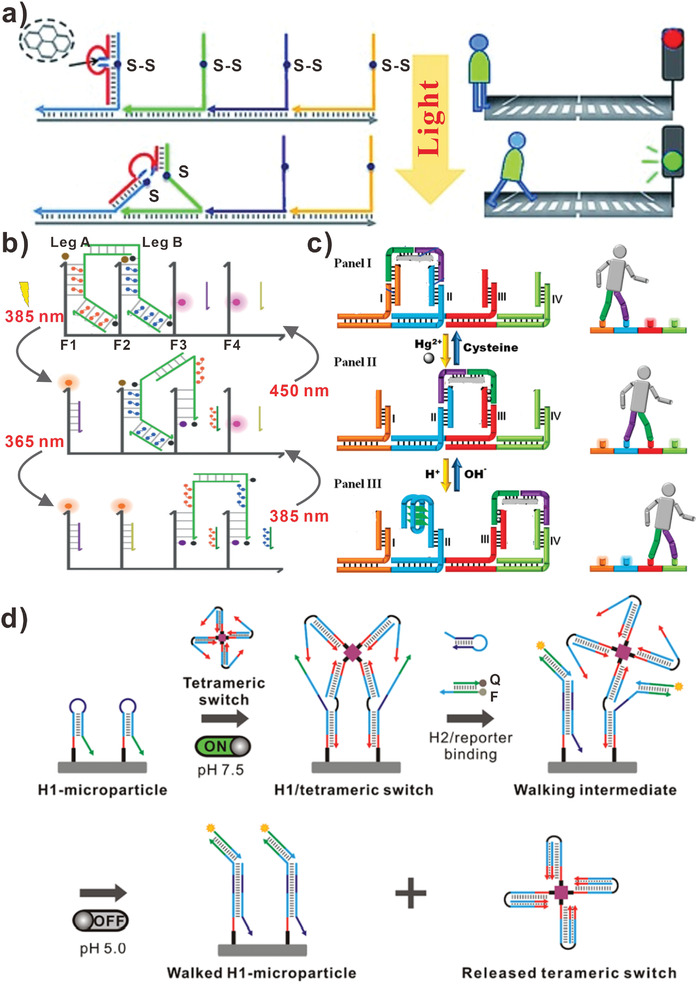
DNA walkers driven by environmental stimulus. a) Light‐driven DNA walkers. Reproduced with permission.^[^ ^66^ ^]^ Copyright 2012, Wiley‐VCH. b) Orthogonally light‐controlled DNA walkers. Reproduced with permission.^[^ ^67^ ^]^ Copyright 2019, Wiley‐VCH. c) H^+^/OH^−^ and Hg^2+^/cysteine stimulus‐activated DNA walkers. Reproduced with permission.^[^ ^68^ ^]^ Copyright 2011, American Chemical Society. d) DNA walkers driven by pH changes. Reproduced with permission.^[^ ^69^ ^]^ Copyright 2020, American Chemical Society.

In addition to strand displacement reactions and enzymatic reactions, environmental stimulus were also introduced to drive DNA walkers. In this section, we will summarize the DNA walkers powered by light and chemical stimulus. It is well‐known that photochemical reaction is initiated by absorbing energy from light.^[^ ^70^, ^71^, ^72^, ^73^ ^]^ In 2012, Tan and his team designed a DNA walker that was capable of initiating, moving, and stopping with the control of light (**Figure** **3**a).^[^ ^66^ ^]^ DNA walkers powered by light energy could be terminated at a desired position or time, and could be restarted easily. In this work, Tan et al. found aromatic hydrocarbons could promote the photolysis of disulfide bonds efficiently. They constructed artificial nucleic acid backbones that contained disulfide bonds. In addition, they designed pyrene‐incorporated DNAzyme analog that could possess catalytic cleavage function. The light‐sensitive DNA walker contained a short leg and a long leg linked by a pyrene moiety. With the light irradiation at 350 nm, the disulfide bond on the stator strand was photolyzed, and the shorter leg was dissociated. Based on the driving force of toehold‐mediated strand displacement, the DNA walker moved to the next stator strand. Due to the fact that the light energy could be transmitted without physical contact, the movement of DNA walker could be achieved through switching of the light power. Further, in order to apply light‐driving DNA walker in more fields, such as nano‐factories, smart materials, and therapeutics, Marko et al. designed orthogonally light‐controlled non‐autonomous DNA walker in 2019 (Figure 3b).^[^ ^67^ ^]^ In this assay, they introduced two azobenzene derivatives, S‐DM‐Azo and DM‐Azo, which enabled a bipedal walker to be controlled by strand displacement reactions in a non‐autonomous way. The strand displacement reactions depended on the wavelength of light. It demonstrated that the non‐autonomous DNA walker could be expanded by introducing another orthogonal wavelength. Later, Liu et al. presented a DNA walker integrated with photo‐controlled module and DNAzyme module for intracellular microRNA imaging.^[^ ^74^ ^]^ Initially, the bipedal DNA walker was silenced by the blocker strand. Then the blocker strand was cleaved by light‐initiated reaction after being irradiated by UV light. With the addition of Mn^2+^, the DNA substrate was cleaved into two fragments by the DNAzyme. Subsequently, the FAM‐labeled fragment was released and emitted fluorescence signals in living cells.

In addition to the light energy, the chemical energy also serves as driving force for DNA walker. A typical example was the chemical energy‐driven DNA walker constructed by Willner and his colleagues in 2010.^[^ ^68^ ^]^ The DNA walkers could be activated by H^+^/OH^−^ and Hg^2+^/cysteine stimulus (Figure 3c). First, there were four footholds stabilized on a DNA template, the DNA walker consisted of two legs hybridized with the former two footholds. Then, treating the system with Hg^2+^ ions, the first leg of DNA walker walked from foothold I to foothold III with the formation of more stabilized T‐Hg^2+^‐T structure. Subsequently, treating the current system with cysteine led to the formation of Hg^2+^‐cysteine complex, and the leg of DNA walker walked back to foothold I again. In turn, treatment of the system with H^+^ ions (pH = 5.2) in panel II resulted in the formation of i‐motif structure on the foothold II, resulting in the DNA walker moving from foothold II to foothold IV. Once the current system was neutralized in panel III, the i‐motif structure was dissociated, thus the DNA walker moved back to foothold II again. As a result, the forward walking was activated by the formation of T‐Hg^2+^‐T structure and i‐motif structure that was forced by Hg^2+^ ions and H^+^ ions, respectively. And the backward walking of DNA walker was activated by the disassociation of i‐motif structure and T‐Hg^2+^‐T complex that was driven by OH^−^ ions and cysteine, respectively. To develop more practical nanomachines, Jung et al. designed a four‐legged DNA walker driven by pH changes (Figure 3d).^[^ ^69^ ^]^ In detail, the DNA walker was comprised of four pH‐responsive CG‐C^+^ triplexes. The activities of DNA walker, such as starting, stopping, walking rate, and steps, could be controlled efficiently by pH changes.

## 3 Number of Legs for DNA Walkers

According to the number of moving legs, DNA walkers can be divided into unipedal DNA walkers, bipedal DNA walkers, and multipedal DNA walkers. Recently, DNA walkers with different numbers of legs have been developed rapidly in various research. Specifically, each type of DNA walkers has its own unique advantages. For instance, unipedal DNA walkers move with high velocity and are easy to design. Bipedal DNA walkers and multipedal DNA walkers show higher stability and longer moving distance. Up to now, the goal of designing next‐generation DNA walker is to increase the processivity, velocity, and directionality of the DNA walkers.^[^ ^11^, ^75^ ^]^

### 3.1 Unipedal DNA Walkers

**Figure 4:**
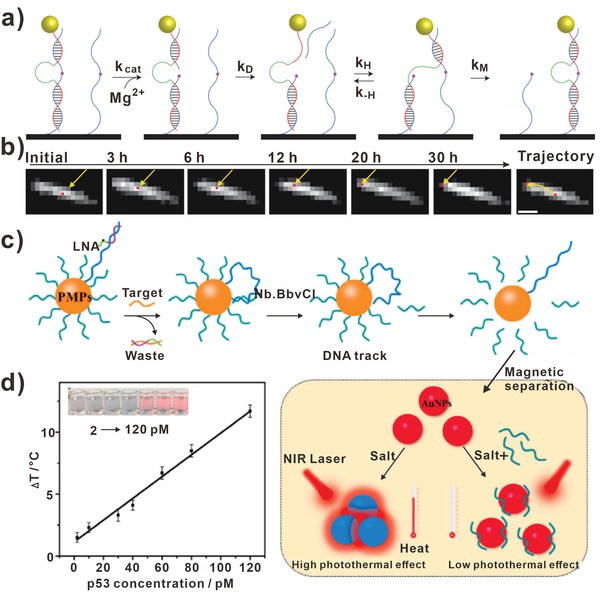
Unipedal DNA walkers. a) Unipedal DNA walkers based on 1D track. b) Images recorded over 30 h of unipedal DNA walker moving, scale bar, 2 µm. (a,b) Reproduced with permission.^[^ ^65^ ^]^ Copyright 2013, Springer Nature. c) Unipedal DNA walkers based on 3D track. d) The relationship between Δ*T* and the concentration of p53 target. (c,d) Reproduced with permission.^[^ ^78^ ^]^ Copyright 2020, Elsevier.

As the name suggests, the unipedal DNA walkers can walk with only one leg. Unipedal DNA walkers can be divided into two types according to different shapes of the walking track. For 1D and 2D walking track, the legs of unipedal DNA walkers always contain two parts. In detail, the relatively short part acts as the free foot moving to the next DNA stator passively, and the other part can be driven to the next DNA stator subsequently. As the previously mentioned DNA walkers constructed by Hyun Choi et al. (**Figure** **4**a), the walking track was a 1D SWCNT absorbing non‐covalently with closely adjacent RNA molecules.^[^ ^65^ ^]^ The unipedal DNA walker was driven by enzymatic reaction, a DNAzyme was attached to the DNA walker. The shorter part of the leg contained 7 bases and the longer part was 16 base in length. In the presence of Mg^2+^, the RNA track was divided into two fragments. Due to that, the cleavage rate *k*_cat_ was greater than the ligation rate *k*_ligation_, the shorter fragment of RNA stator could dissociate from the carbon nanotube. Then the unpaired, shorter leg of DNA walker bound to the next adjacent RNA stator and the following strand replacement reaction occurred. Therefore, a more thermodynamically stable state was transited with the first step of unipedal DNA walker accomplished. By introducing Mg^2+^ continuously, the unipedal DNA walker walked along 1D carbon nanotube directionally. In Figure 4b, optical images of the DNA walker moving along a nanotube were shown. The red spots indicated the position of the nanoparticle. Obviously, the nanoparticle moved from the center to the left edge of nanotube. As shown by the yellow arrows in the rightmost panel, the DNA walker traveled 3 µm in 30 h.

To guarantee the longer time for moving, Jung and colleagues designed a cleated DNA walker hanging on to the surfaces of streptavidin (SA)‐coated microparticles in 2017.^[^ ^76^ ^]^ The unipedal DNA walker had a “cleat” at end of the leg, which allowed it to associate with the DNA track persistently. As shown by the movement over the microparticle surface, the unipedal DNA walker could proceed over long periods of time. In addition, there were many researches constructing unipedal DNA walkers on 2D surfaces, such as DNA origami. For example, Wang and colleagues presented a DNA walker that moved on the DNA origami surface.^[^ ^77^ ^]^ It is worth noting that the DNA target served as unipedal DNA walker for fluorescent signal amplifying. First, DNA target (DNA walker) bound to the DNA origami track by hybridizing with stator strands. Then, nicking enzyme cleaved DNA duplex, exposing sticky end to combine with imager strand. With the moving of DNA walker, more and more imager strand could be combined, accomplishing the simplification of fluorescent signal.

On the 3D track, the other type of unipedal DNA walker possesses a long, intact walking leg with one end fixed on surface of 3D track while the other end drives freely on the track. The typical 3D track is AuNPs and other microparticles. As the previously mentioned FEN 1‐assisted DNA walkers by Cheng et al.^[^ ^49^ ^]^ The target DNA was stabilized on the AuNP, which walked on the surface of the AuNP by hybridizing with the DNA track strands. FEN 1 was employed to recognize the duplexes and cleave the overlapping structure. Then the fluorescent tags were released and the system generated amplified signals. Other than quenching fluorescent signals, AuNPs can be used to produce photothermal effect. Tao and his colleagues designed a DNA walker biosensor for p53 sensing and applied a thermometer as the readout (Figure 4c).^[^ ^78^ ^]^ Similarly, the DNA walker was anchored on the surface of AuNP, locked nucleic acid (LNA) was functionalized on the end of DNA walker. Once the p53 was introduced, it could hybridize with the LNA and simultaneously initiate the startup of DNA walkers. With the spontaneous walking of DNA walker along the AuNPs, the DNA track was cleaved. As a result, the short DNA coated‐AuNPs were produced, which resisted the aggregation of AuNPs. When lasered by NIR, the light‐to‐heat conversion decreased due to the fact that AuNPs could not aggregate in the solution. In this assay, the detection of p53 was achieved by evaluating the temperature change value. As illustrated in Figure 4c, the changes of temperature were positively related to the concentration of p53. It is worth noting that the photothermal biosensor exhibited a low LOD of 1.4 pM for the detection of p53 without any advanced analytical instruments.

### 3.2 Bipedal DNA Walkers

**Figure 5:**
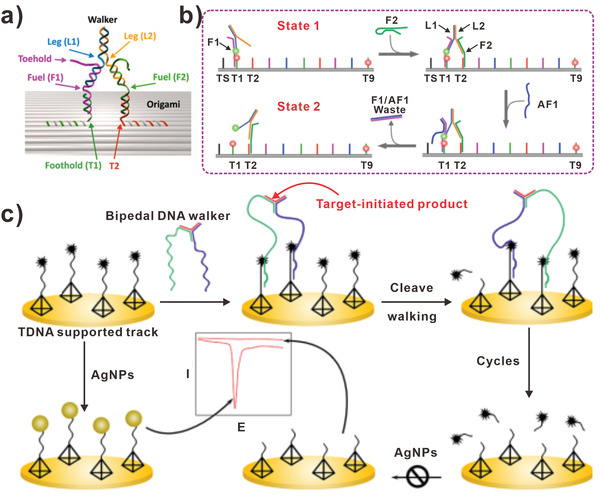
Bipedal DNA walkers. a) Bipedal DNA walkers based on 2D track. b) The principle of DNA walker operation. (a,b) Reproduced with permission.^[^ ^79^ ^]^ Copyright 2017, American Chemical Society. c) Bipedal DNA walkers based on the electrochemical strategy. Reproduced with permission.^[^ ^80^ ^]^ Copyright 2019, American Chemical Society.

For morphological perspective, the bipedal DNA walkers have two relatively free legs to walk alternately. Theoretically, they would walk along the DNA tracks for a longer time, achieving higher signal amplification efficiency. Recently, strand displacement reactions and enzymatic reactions have been used widely in developing bipedal DNA walkers. For instance, Eyal and his team constructed a bipedal DNA walker which accomplished a large number of steps (**Figure** **5**a).^[^ ^79^ ^]^ In detail, the bipedal DNA walkers were composed of two legs (L1 and L2) that moved on a DNA origami. As shown in Figure 5b, the DNA track on origami had four different sequences. In addition, DNA legs were connected on the DNA track by hybridizing with fuel strands (F1‐F4) and were disconnected by the strand displacement reaction initiated by antifuel strands (AF1‐AF4). Furthermore, 64 consecutive commands were input to the bipedal DNA walker, driving the DNA walker to move 32 steps on DNA origami. Notably, the microfluidics enabled programmable control of the direction and speed of the bipedal DNA walkers. Electrochemical biosensors are used widely with measurable electrical signals produced by the chemical reactions between the targets and recognition elements. In 2019, Liu et al. constructed an electrochemiluminescent (ECL) biosensor based on DNA walker for ultrasensitive detecting microRNA‐21 (miRNA‐21).^[^ ^81^ ^]^ In this system, the movement of bipedal DNA walker was triggered by miRNA‐21 and powered by following strand displacement reactions for signal amplification. For the electrochemical signal producing, amino‐modified 3,4,9,10‐perylenetetracarboxylic dianhydride/luminol (PTC‐NH_2_/Lu) nanocomposite was used as signal producer. With the presence of miRNA‐21, bipedal DNA walkers were formed. Then, PTC‐NH_2_/Lu probes were captured on the GCE surface and output ECL signals. Therefore, the integration of bipedal DNA walker and ECL provided a novel signal amplification platform for early clinical diagnostics.

Enzymatic reactions have been used most widely in constructing bipedal DNA walkers. For instance, Chai and colleagues also developed a bipedal DNA walkers based on the electrochemical strategy (Figure 5c).^[^ ^80^ ^]^ They synthesized magnetic Fe_3_O_4_@gold nanoparticles (Fe_3_O_4_@AuNPs) for enriching target DNA. In detail, low abundant target DNA could hybridize with probe A that stabilized on the Fe_3_O_4_@AuNPs. After DNA polymerizing, nicking, and strand displacing, a large number of strands (probe B) were released and enabled the probes C and D to get closely. Furthermore, probes C and D could be regarded as two legs of a bipedal DNA walker. Due to that probes C and D contained metal ion‐dependent DNAzyme tail sequence, the DNA tracks anchored on the tetrahedron DNA were cleaved in the presence of Pb^2+^. Meanwhile, the DNA walker explored the neighboring intact track and continued walking process. Due to that the DNA tracks were modified with amino groups for adsorbing AgNPs as electrochemical probes, the cleaving of DNA tracks brought about decreased electrochemical signals. Consequently, the target could be quantitatively analyzed by measuring the electrochemical signals. Obviously, it is more reliable for electrochemical system to apply “signal on” strategy than “signal off” strategy. For instance, Zhu et al. designed an ultrasensitive photoelectrochemical (PEC) biosensor based on bipedal DNA walker.^[^ ^82^ ^]^ In this work, bipedal DNA walker was used to change the distance of ferrocene (Fc) and methylene blue (MB) to the photoactive material perylene‐3,4,9,10‐tetracarboxylic acid (PTCA), which originally showed a high PEC signal. Upon the Fc labeled hairpin DNA 1 (H1‐Fc) anchored on the surface, a significant signal reduction occurred. However, in the presence of target thrombin, the H1‐Fc was opened and left Fc away from PTCA, achieving the recovery of PEC signal. Finally, the exposed toehold was hybridized with hairpin DNA 2 labeled with MB (H2‐MB). The approaching of MB molecules to PTCA realized the “signal on” process. In any case, the “signal on” strategy provided an efficient avenue for sensitive detecting biomolecules.

### 3.3 Multipedal DNA Walker

**Figure 6:**
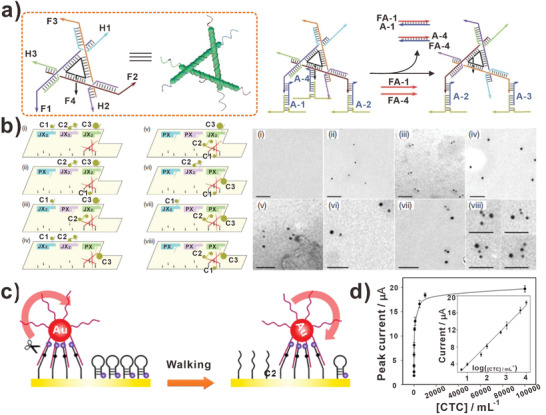
Multipedal DNA walkers. a) Typical DNA walkers with three hands and four legs. b) Schematics of the final state for the DNA walker system and corresponding transmission electron microscope images of the products. Scale bars, 50 nm. (a,b) Reproduced with permission.^[^ ^47^ ^]^ Copyright 2010, Springer Nature. c) AuNPs‐based multipedal DNA walker. d) Relationship between peak current and the target CTC concentration. (c,d) Reproduced with permission.^[^ ^83^ ^]^ Copyright 2019, American Chemical Society.

To improve the performance of biosensors based on DNA walkers, researchers have studied many forms of multipedal DNA walkers. Multipedal DNA walkers mainly include two types, one is that DNA walker with certain number of legs, and the other is DNA walker with a large number of legs. A typical example for the former is the multipedal DNA walker designed by Gu et al. (**Figure** **6**a).^[^ ^47^ ^]^ The multipedal DNA walker transferred three types of AuNPs, and produced eight different products. In detail, the DNA walker contained three hands and four legs. The hands could bind cargos and the legs could hybridize with single‐strands on the DNA track. Specifically, the fourth legs of the multipedal DNA walkers were bound at stations where cargos to be placed, which ensured the proper orientation of cargos. The key advantage of the system was the high programmability of the cargo‐donating multipedal DNA walkers, which allowed to generate eight different products. Figure 6b presented the schematics of the final products and corresponding transmission electron microscope images of the products. Another example was a circular DNA walker consisting of four footholds envisaged by Wang et al.^[^ ^68^ ^]^ To visualize the motion of DNA walker, the fluorophore‐labeled strands hybridized with footholds respectively. The DNA track could be bound to the different footholds with sticky ends at different conditions (Hg^2+^/cysteine and H^+^/OH^−^). And the state of the DNA walker on the circle could be read out by the characteristic quenching fluorophore. In conclusion, the triggers enabled the forward and backward walking of the DNA walkers. Furthermore, the application of such DNA walkers may provide a chance to sense ions in cells. In order to detect proteins, Li and colleagues developed chemiluminescence biosensors based on catalyzed hairpin assembly reaction (CHA) and isothermal strand‐displacement polymerase reaction.^[^ ^86^ ^]^ The multipedal DNA walker, constructed by a biotin‐modified catalyst, could interact with the DNA track on the magnetic microparticle (MMPs) and open the hairpin structure. Then, the opened structure was hybridized with biotin‐labeled H2. Based on the strand displacement process, one leg of the DNA walker was released and continued to interact with the next DNA track. Using this multipedal DNA walker, streptavidin could be detected with the limit of 6.5 pM. Furthermore, the DNA walker was applied to detect folate receptor and thrombin successfully.

In addition, it is proved that the walking rate of multipedal DNA walkers was much faster than unipedal and bipedal DNA walkers. Therefore, a series of multipedal DNA walkers based on nanoparticles have been developed. For example, Miao et al. constructed AuNPs‐based multipedal DNA walker for detecting CTCs (Figure 6c).^[^ ^83^ ^]^ First, a large amount of walker strands integrated with aptamer sequence were modified on AuNPs. Then the aptamer specifically interacted with the receptor protein of CTCs. Hence the CTCs could be precipitated after centrifuging, and the supernate containing a small amount of AuNPs presented decreased UV–vis absorbance of AuNPs. Furthermore, the multipedal DNA walkers in supernate hybridized with DNA tracks on the surface of electrodes, and the DNA tracks were cleaved by nicking endonuclease, leading to the increase of electrochemical signal. Figure 6d showed the relationship between electrochemical signal and CTC concentration. By combining the changes of UV–vis absorbance and electrochemical signal, this multipedal DNA walker achieved ultrahigh sensitivity (1 cell mL^−1^) for CTCs detection. Similarly, Miao et al. also designed a multipedal DNA walker based on magnetic Fe_3_O_4_@Au nanoparticle in 2020.^[^ ^87^ ^]^ As the designing principle illustrated, the multipedal DNA walker was formed based on the Fe_3_O_4_@Au nanoparticle. In detail, the Fe_3_O_4_@Au nanoparticles were modified with CD63 aptamer sequence. Due to the fact that CD63 aptamer specifically recognized CD63 proteins on Hela cell‐derived exosomes, the Fe_3_O_4_@Au nanoparticles could be conjugated with exosomes. Then probes B were added and conjugated to the surface of exosomes. Subsequently, the multipedal DNA walkers were developed. After magnetic separation to remove redundant unconjugated probes B, probes C and nicking endonuclease were introduced to cleave the linker between Fe_3_O_4_@Au and exosomes. Then the second magnetic separation was carried out, after which multipedal DNA walkers were purified. The walking track was stabilized on the surface of electrode, which led to the production of electrochemical signals. In this study, the exosome level could be quantitatively evaluated with the detection sensitivity of 6/µL.

**Figure 7:**
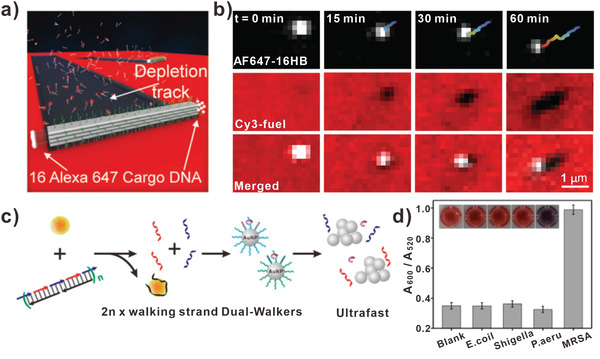
Multipedal DNA walkers. a) DNA origami‐based multipedal DNA walker. b) The time‐lapse fluorescence images of the DNA walker and the fuel strains. (a,b) Reproduced with permission.^[^ ^84^ ^]^ Copyright 2020, Wiley‐VCH. c) Dual DNA walkers for rapid detecting bacteria. d) The absorbance of the colorimetric biosensor with different targets. Inset: corresponding photographs. (c,d) Reproduced with permission.^[^ ^85^ ^]^ Copyright 2020, American Chemical Society.

In addition, to achieve a large number of steps of multipedal DNA walkers, Ke and his team designed a highly tunable DNA origami walker that moved over micron distance (**Figure** **7**a).^[^ ^84^ ^]^ Importantly, the multipedal DNA walker moved at an average speed of 40 nm min^−1^. Due to that DNA origami can be designed precisely in shape, size, and the density of legs, researchers constructed origami‐based DNA walker with optimal design. The multipedal DNA walker consisted of rectangular prism origami and 36 legs on each face of the rectangular prism (total = 144). Furthermore, 8 cargo‐binding DNA strands were loaded at each end of the origami (total = 16) with AF647‐tagged strands and the track was composed of single‐stranded RNA (ssRNA) tagged with Cy3, enabling to track the motion of DNA walker. Figure 7b showed the fluorescent images of A647‐16HB, Cy3‐fuel, which revealed the DNA origami‐based DNA walker moved across the RNA tracks. In the field of nano‐optics, it is challenging to transport optical nano‐object to a predefined destination along the programmed path. As a result, Liu et al. developed an active plasmonic system based on the multipedal DNA walker, in which the gold nanorod (AuNR) could execute controllable movement on the 2D or 3D origami.^[^ ^45^ ^]^ The DNA walker comprised an anisotropic gold nanorod and discrete DNA strands. In addition, the DNA walker carried optical information with the presence of AuNR. Therefore, the walking directions and steps could be optically reported at nanometer accuracy. Detailly, the walking system comprised a walker and a stator. As a consequence of the movement of DNA walker, plasmonic coupled system changed its own structure. Subsequently, the near‐filed interaction of AuNR adjusted, which could be read out optically.

Recently, to optimize the process of biosensing, Pei and his team designed DNA dual‐walkers for detecting bacteria (Figure 7c).^[^ ^85^ ^]^ The DNA dual‐walkers were different from bipedal DNA walkers. Conversely, DNA dual‐walkers were more like the mixture of two unipedal DNA walkers moving on DNA tracks, respectively. First, two kinds of thiol‐tagged strands were functionalized on the surface of AuNPs, forming two types of DNA track. Then, the two kinds of DNA walkers were blocked with the aptamers of target bacteria. In the presence of target bacteria, the aptamers (black strands) bound to targets and two kinds of DNA walkers (blue and red strands) were released. Based on the enzymatic reaction, the DNA dual walkers could walk on the AuNP‐based 3D track, resulting in the aggregation of AuNPs. It can be seen from Figure 7d, the colorimetric biosensor presented superior selectivity of the target MRSA. Furthermore, the dual walkers‐based biosensors showed sensitive color changes and provided a simple tool for ultrafast colorimetric bacteria detection.

## 4 DNA Walkers‐Based Biosensors

A variety of biomarkers have been used widely in diagnosing diseases in recent years.^[^ ^88^, ^89^, ^90^ ^]^ Therefore, it is vital to develop various biosensors capable of sensing biomarkers with low detection limit and high specificity.^[^ ^2^, ^8^, ^91^, ^92^, ^93^ ^]^ To improve the sensitivity of biosensors, a variety of strategies have been raised to amplify signals, such as enzymatic reactions, DNA nanotechnology, and so on.^[^ ^94^, ^95^ ^]^ Among them, DNA walker‐based biosensors exhibit superior performance in biosensing. The programmability of DNA nanotechnology enables the design of walking behaviors that specifically respond to different biological targets. Besides, the DNA walkers can walk along the designed tracks spontaneously, leading to the powerful signal amplification. In this section, we will summarize the current research in DNA walker‐based biosensors for various targets analysis. Then how to rationally design DNA walkers to construct sensitive biosensors is discussed.

### 4.1 DNA Walkers for Nucleic Acids Detection

**Figure 8:**
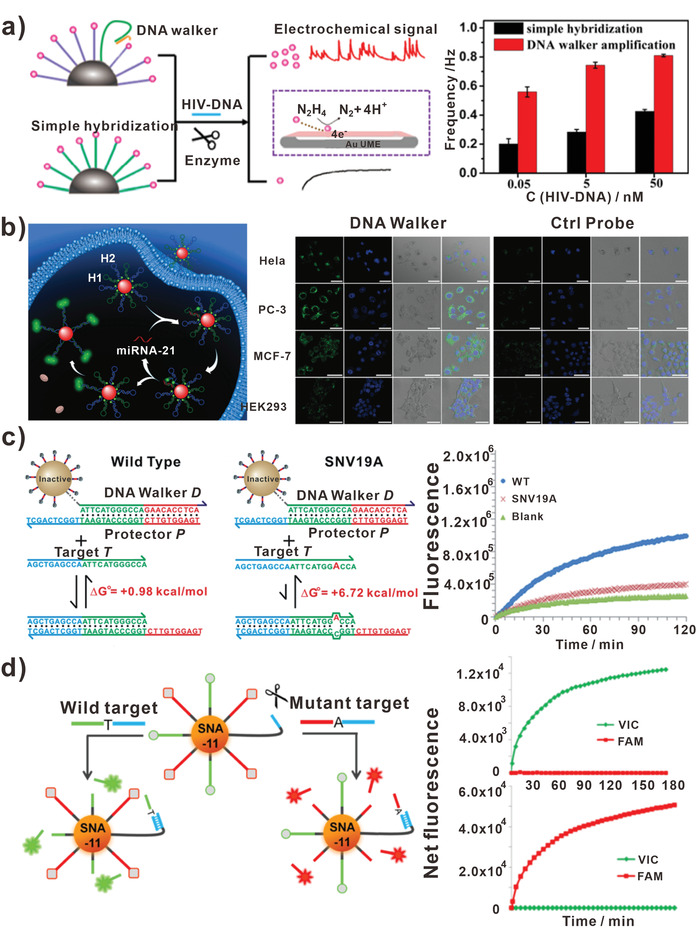
DNA walkers‐based nucleic acids biosensors. a) DNA walkers for biosensing HIV‐DNA. Reproduced with permission.^[^ ^96^ ^]^ Copyright 2021, American Chemical Society. b) DNA walkers for imaging miRNA. Scale bar: 50 µm. Reproduced with permission.^[^ ^97^ ^]^ Copyright 2020, Royal Society of Chemistry. c) DNA walkers for detecting SNV in DNA target. Reproduced with permission.^[^ ^98^ ^]^ Copyright 2018, Royal Society of Chemistry. d) DNA walkers for detecting SNV in ctDNA target. Reproduced with permission.^[^ ^49^ ^]^ Copyright 2021, American Society Chemistry.

Nucleic acids (DNA and RNA) are important carriers that store, delivery, and express information of organisms.^[^ ^99^, ^100^ ^]^ The sequences and concentrations of nucleic acids have been strongly demonstrated to be implicated in many disorders‐related diseases.^[^ ^101^, ^102^ ^]^ Therefore, the detection of nucleic acids is extremely significant. For example, viruses, the collection of nucleic acids, can infect cells and kill the host cells and cause huge damage to organism. The viruses can be classified into two categories: one is RNA viruses, such as SARS, MERS, COVID‐19, and Ebola viruses, the other is DNA viruses, including HIV, HBV, and so on. Therefore, the nucleic acids in viruses are the important target for clinical diagnosis. Wang and colleagues designed DNA walker‐based fluorescent biosensor with localized CHA (LCHA) for detecting Zika viruses (ZIKV).^[^ ^103^ ^]^ In this work, the DNA walker and hairpin DNA tracks were immobilized on the AuNPs. And the DNA walker was locked on the other end. In the presence of ZIKV RNA, the DNA walker was unlocked and moved along the AuNPs, cleaving the DNA tracks and generating numerous trigger sequences. Then LCHA was activated and fluorescence signal was generated. Eventually, the DNA walker‐based biosensors allowed the detection limit of ZIKV RNA down to 20 pM. Similarly, Jiang et al. also constructed a 3D DNA walker‐based biosensor for detecting Ebola viruses.^[^ ^104^ ^]^ First, the DNA walker was immobilized on the surface of AuNPs. When Ebola RNA presented, the DNA walker was released and cleaved DNA tracks at the aid of Exo III. For Ebola viruses RNA detection, the detection limit can be as low as 3.5 fM with excellent specificity. Other than fluorescence, electrochemical signal can also be used to detect viruses. Wang et al. developed an electrochemical biosensor based on DNA walker for human immunodeficiency virus DNA (HIV‐DNA) counting (**Figure** **8**a).^[^ ^96^ ^]^ Different from the former studies, the introduction of HIV‐DNA could lead to the release of PtNPs that were immobilized on the DNA tracks. Then the PtNPs generated collision current transients, which was related to the concentration of targets. In this assay, about threefold collision frequency could be produced with the help of DNA walker system. The detection limit can be as low as 4.86 fM, which was far lower than traditional biosensors.

Apart from viral nucleic acids, ‐miRNA‐, known as an important biomarker, is found to be critical for monitoring physiological environment. Le and colleagues designed a microRNA‐initiated DNAzyme walker operated in living cells.^[^ ^105^ ^]^ The DNA walker system was developed on a 20 nm AuNP decorated with hundreds of DNA strands and a silenced DNAzyme molecule. When miRNA target interacted with the DNA walker system in cell, it then initiated the autonomous walking of DNA walker on the AuNP. In this assay, the DNA walker accomplished 30 walking steps within 30 min. Similar DNA walker system could be designed to respond to other messenger RNA targets. When altered the target binding domain on the locking strand, DNA walker system could also be developed to respond to proteins and small molecules in cells. Xu et al. raised an enzyme‐free 3D DNA walker for illuminating miRNA (Figure 8b).^[^ ^97^ ^]^ In this assay, AuNPs were used as carriers of DNA hairpins as well as quenchers of fluorescence. Furthermore, two kinds of FAM‐labeled DNA hairpins (H1 and H2) were decorated on AuNP. In the presence of miRNA‐21, the H1 hairpin structures were opened. Then H1 interacted with H2 to form a more stable duplex and released miRNA‐21. In Figure 8b, the live‐cell images of miRNA‐21 with DNA walker and control probe in Hela, MCF‐7, PC‐3, and HEK293 cells were presented in the right panel. Based on the catalytic amplification strategy, the limit of detection (LOD) of miRNA‐21 was decreased to 6.1 pM.

Due to that mutation of DNA target leads to potential biological and clinical concerns, the single nucleotide variants (SNV) are important biomarkers for clinical diagnosis. Other than intact DNA sequence, the mutation of DNA target should also be detected. Li and colleagues designed nicking endonuclease‐powered 3D DNA walkers for discriminating SNV (Figure 8c).^[^ ^98^ ^]^ Initially, the DNA swing leg was blocked by an affinity ligand. In the presence of target, the binding of the DNA target brought the swing leg to DNA track. Then the movement of the swing leg around the AuNP surface was initiated. Subsequently, the swing leg moved along DNA track and cleaved hundreds of DNA strands in response to the single binding event. Specifically, the researchers introduced an auxiliary probe to release targets into solution, which enabled to construct biosensors with high sensitivity and specificity. In Figure 8c, the fluorescent responses of DNA walker‐based biosensors to 1 nM WT and SNV were measured. It was obvious that the fluorescent curve of WT was higher than SNV. The biosensors based on 3D DNA walkers improved the sensitivity by ∼100 times. As mentioned earlier, the DNA walkers designed by Cheng et al. could discriminate SNV of ctDNA targets (Figure 8d).^[^ ^49^ ^]^ The FEN 1 assisted‐DNA walker presented excellent performance in achieving SNV detection. In addition, the right fluorescent curves in Figure 8d were signals of DNA walker for the detection of 100 pM wild type (top) and mutant (bottom) target DNAs. As illustrated in this assay, the detection limits for DNA target and mutation abundance reached 0.22 fM and 0.01%, respectively. The consistent results between DNA walker‐based biosensors and the next‐generation sequencing demonstrated the potential of DNA walker in liquid biopsy.

### 4.2 DNA Walkers for Proteins Detection

**Figure 9:**
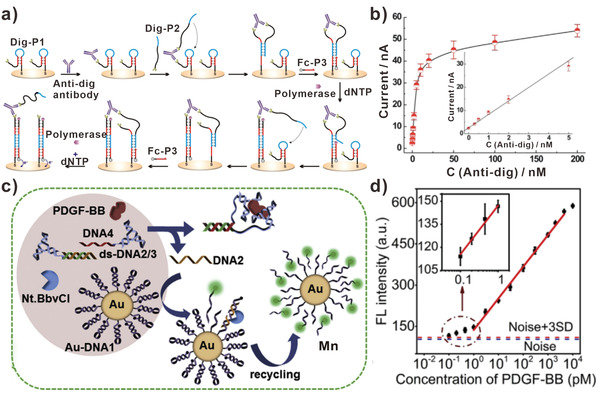
DNA walkers‐based proteins biosensors. a) DNA walkers for biosensing digoxin. b) Calibration curve between electrochemical signal and anti‐dig antibody concentration. (a,b) Reproduced with permission.^[^ ^106^ ^]^ Copyright 2019, Elsevier. c) DNA walkers for biosensing PDGF‐BB. d) The relationship between fluorescent intensity and PDGF‐BB concentration. (c,d) Reproduced with permission.^[^ ^107^ ^]^ Copyright 2019, Elsevier.

At present, rapid and sensitive detection of proteins plays an important role in early diagnosis of diseases.^[^ ^110^, ^111^, ^112^, ^113^ ^]^ However, it is still lacking PCR‐like amplification techniques for proteins. It is still challenging in analyzing proteins with low abundance. Recently, the development of DNA walker strategies can help to solve this problem effectively. For instance, Liu et al. developed polymerase‐powered DNA walker for protein analysis with amplified electrochemical signals (**Figure** **9**a).^[^ ^106^ ^]^ The DNA walker strategy was proposed on the basis of protein binding‐induced proximity recognition and polymerase amplification. The hairpin‐like DNA tracks were immobilized on the electrode, the other ends of the strands were decorated with protein recognition elements. Interestingly, the target protein bound with DNA track brought about the proximity hybridization with DNA walker as well. In the presence of polymerase, the primer strand was polymerized and DNA walker was released, which then moved to the adjacent DNA track for the next operation. The sensing performance of DNA walker‐based biosensors for anti‐dig antibody was explored in Figure 9b. Using anti‐dig antibody and biotin as recognition elements, the detection limits of digoxin and SA were 80 and 16 pM, respectively. Based on the wide study of protein recognition elements (antibody, aptamer, etc.), the protocol in this assay offered a general and simple strategy for protein quantification. Thanks to the proximity hybridization‐induced signal amplification, Ju and his team proposed a DNA walker for ultrasensitive detecting platelet‐derived growth factor (PDGF‐BB) (Figure 9c).^[^ ^107^ ^]^ Unlike previous studies, DNA walkers in this work were activated by the specific binding of PDGF‐BB with aptamers. Then, the free DNA walkers moved along the DNA tracks, resulting in the release of FAM decorated fragments. As shown in Figure 9d, this strategy could detect PDGF‐BB secreted by cancer cells with sub‐pM level detection limit.

**Figure 10:**
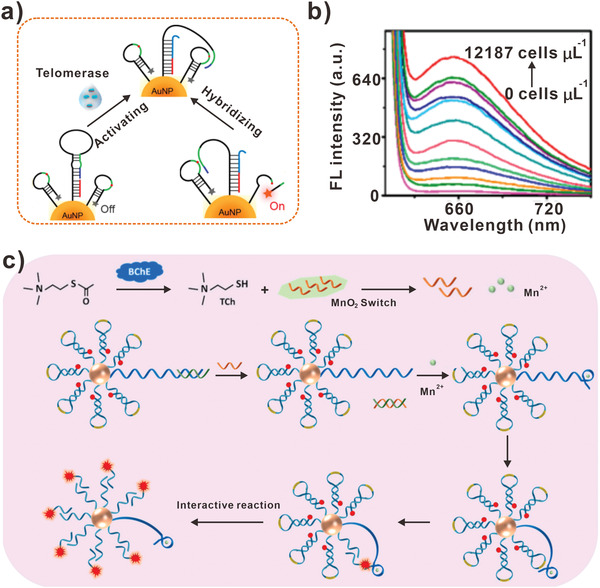
DNA walkers‐based protein biosensors. a) DNA walkers for detecting human telomerase activity. b) The fluorescent intensity of DNA walker incubated with different Hela cells concentration. (a,b) Reproduced with permission.^[^ ^108^ ^]^ Copyright 2019, American Chemical Society. c) DNA walkers for detecting the activity of ChE. Reproduced with permission.^[^ ^109^ ^]^ Copyright 2020, Elsevier.

Other than protein targets, the activity of enzymes is also regarded as an important parameter in evaluating biological process. Lei et al. constructed a DNA walker with super‐hairpin structure for sensing human telomerase activity (**Figure** **10**a).^[^ ^108^ ^]^ The bulged loop in the super‐hairpin served as walking leg. And the primers in super‐hairpin were elongated with the catalysis of telomerase, which triggered the opening of loop structure. Subsequently, released DNA walker hybridized with the track and the generated duplex could be cleaved by enzymes, resulting in the production of fluorescent signal. In this assay, the detection limit of telomerase activity for Hela cells was equivalent to 90 cells µL^−1^ (Figure 10b). Recently, Wang et al. designed MnO_2_ switch‐bridged DNA walker for sensing the activity of cholinesterase (ChE) (Figure 10c).^[^ ^109^ ^]^ In this design, the activity of ChE was converted to the running of DNA walker. With the action of ChE, the MnO_2_ nanosheet was reduced to Mn^2+^ and the fuel strands were released. Then the movement of DNA walker was triggered by toehold mediated strand displacement of fuel strands at the aid of Mn^2+^. In addition, the amplifying fluorescent signal could reflect the activity of ChE. Benefited from the amplification effect of DNA walker, the detection limitation as low as 0.0008 U mL^−1^ was achieved. Therefore, DNA walkers represented a powerful tool for biosensing protein targets and the activity of enzymes.

### 4.3 DNA Walkers for Other Targets Detection

**Figure 11:**
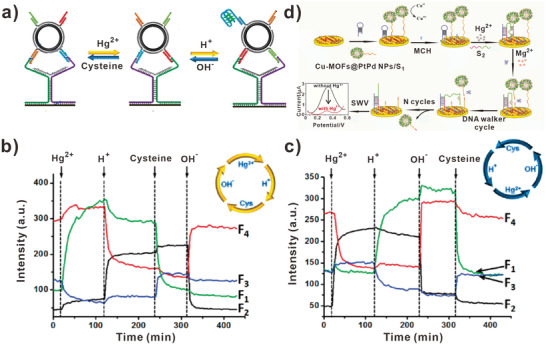
DNA walkers‐based ions biosensors. a) DNA walkers for biosensing H^+^ and Hg^2+^. b) The changes of fluorescent intensity upon activating DNA walkers in the clockwise direction. c) The changes of fluorescent intensity upon activating DNA walkers in the anticlockwise direction. (a‐c) Reproduced with permission.^[^ ^68^ ^]^ Copyright 2011, American Chemical Society. d) DNA walkers for biosensing Hg^2+^. Reproduced with permission.^[^ ^115^ ^]^ Copyright 2021, Elsevier.

So far, a series of DNA walkers have been successfully developed for sensing a variety of biological functions‐related ions. In 2011, Willner and colleagues designed bipedal DNA walkers that were activated by H^+^/OH^–^ and Hg^2+^/cysteine triggers (**Figure** **11**a).^[^ ^68^ ^]^ The bipedal DNA walker was activated on the DNA track consisting of four footholds. In the process of DNA walking, the conformation alterations of thymine‐Hg^2+^‐thymine complex and the i‐motif structure were served as the DNA translocation driving forces. Therefore, the concentration of Hg^2+^ ions and H^+^ ions could be quantified using this strategy. In Figure 11b,c, the dynamic changes of fluorescent intensity were shown. For the first time, Yuan et al. engineered click chemistry reaction to construct DNA walkers for sensing copper ions.^[^ ^114^ ^]^ First, alkynyl‐S1 was immobilized on magnetic polystyrene microsphere@gold nanoparticles (PSC@Au). In the presence of target Cu^2+^, click chemistry reaction occurred and cleaved azido‐S2 to bind with alkynyl‐S1. Then, the hairpin‐locked DNAzyme was opened and cleaved the self‐strand. Therefore, the strand S3 was released. Eventually, the liberated S3 induced CHA reaction on the electrode, which brought the immobilization of MB‐tagged strands and the production of electrochemical responses. Combining click chemistry reaction with the DNA walker amplification strategy, the biosensor showed excellent performance for detecting Cu^2+^ with low detection limit of 0.33 pM. Furthermore, the elaborated biosensor also could be expanded to detect Mg^2+^ and other ions. Recently, Wu and colleagues constructed metal organic frameworks (MOFs)‐based DNA walkers to detect Hg^2+^ (Figure 11d).^[^ ^115^ ^]^ First, Cu‐MOFs@PtPd NPs grown in situ and were labeled with S_1_. Then the hairpin strands and Cu‐MOFs@PtPd NPs/S_1_ were immobilized on the electrode, which produced a larger initial electrochemical signal. In this assay, the designed DNA walker S_2_ contained two sequences. One was Mg^2+^‐dependent DNAzyme that could hybridize with Cu‐MOFs@PtPd NPs/S_1_, and the other was a complementary strand to hairpin DNA track. In the presence of target Hg^2+^, hairpin DNA track hybridized with DNA walker (S_2_) to form a stable structure thymine‐ Hg^2+^‐ thymine (T‐ Hg^2+^‐T). Next, the DNA walker could cleave Cu‐MOFs@PtPd NPs/S_1_ in the presence of Mg^2+^, resulting in the decrease of electrochemical signal. Consequently, the current was negatively related to the Hg^2+^ concentration with a detection limit of 0.52 pM.

**Figure 12:**
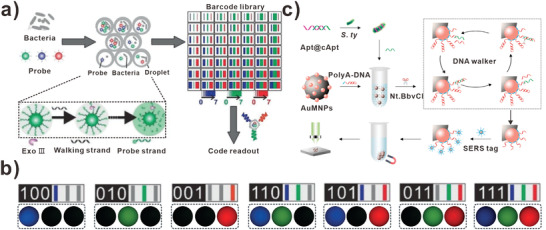
DNA walkers‐based bacteria biosensors. a) SDwalkers‐based barcode library for analyzing multiplex bacterial phenotype. b) The images of multiple barcodes recorded in three channels (blue, green, and red), respectively. (a,b) Reproduced with permission.^[^ ^116^ ^]^ Copyright 2019, Wiley‐VCH. c) DNA walkers for detecting *S. typhimurium*. Reproduced with permission.^[^ ^117^ ^]^ Copyright 2021, Elsevier.

For sensing human health‐related bacteria, DNA walkers also displayed excellent performances. For instance, Pei and his team designed a stochastic DNA walker (SDwalker) for super‐multiplexed bacteria phenotype detection.^[^ ^116^ ^]^ As illustrated in **Figure** **12**a, DNA strands functionalized AuNP served as a 3D DNA track. When the bacteria presented, the aptamer bound to target bacteria, releasing the walking strand. At the aid of Exo III, the DNA walker moved along the DNA track, leading to fluorescence enhancement from the initial quenched state. Especially, the SDwalkers were labeled with three different fluorophores, including AMCA, FAM, TR. Based on a 3‐color/8‐intensity scheme, (8^3^ − 1 = 511) theoretical code could be generated. The fluorescent intensity and images showed seven typical barcodes corresponding to different bacteria (Figure 12b). This system achieved the detection and identification of 20 distinct patterns for bacterial phenotype. Moreover, Pei et al. also developed stochastic DNA dual‐walkers for colorimetric bacteria detection.^[^ ^85^ ^]^ Especially, the dual‐walkers released two kinds of DNA walking strands for moving on DNA track, resulting in the aggregation of AuNPs. Therefore, the change of color from red to blue could be used to analyze the concentration of target bacteria. Because of the fast reaction kinetics and color change, the DNA dual‐walkers could detect bacteria with excellent sensitivity and specificity. In this assay, the biosensors exhibited the liner response ranging from 100 to 105 CFU mL^−1^, and the detection limit reached 1 CFU mL^−1^. Based on the above two examples, the SDwalkers showed promising potential for rapid, specific bacteria detection. For sensitive and efficient detecting *E. coil*, Zheng and colleagues constructed DNA walkers combined with rolling circle amplification (RCA) to amplify electrochemical signal.^[^ ^118^ ^]^ First, the target was extracted from *E. coil* O157:H7 and was amplified by the DNA walker. Then, the RCA reaction was used to amplify the transformed DNA strands, which subsequently triggered the HCR reaction. Based on the multiple amplification strategy, the detection limit of 7 CFU mL^−1^ was achieved. Other than electrochemical strategy, optical strategy also is used to sense bacteria because of the inherent strengths of spectral data, such as surface‐enhanced Raman scattering (SERS). Recently, Duan et al. developed a novel strategy based on DNA walker for quantitative analysis of *Salmonella typhimurium* (*S. typhimurium* ) (Figure 12c).^[^ ^117^ ^]^ Triggered by enzymatic reaction, the DNA walker moved along DNA track. Subsequently, the DNA residues left on the AuMNPs bound to SERS tags and were separated from the solution for SERS analysis. The SERS strategy‐based DNA walker showed excellent LOD as low as 4 CFU mL^−1^. This study presented a novel strategy for SERS detection of bacteria with outstanding sensitivity and selectivity.

### 4.4 DNA Walkers for Single‐Molecule Detection

**Figure 13:**
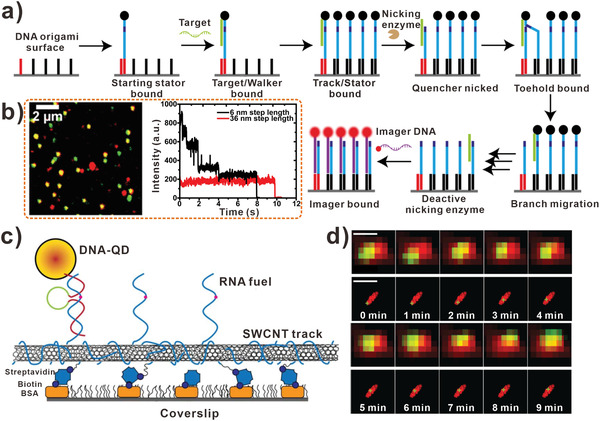
DNA walkers‐based biosensors for single‐molecule detection. a) Fluorescence signal amplification mechanism of DNA walker. b) Image of DNA walker. Green spots indicate origami position; red spots indicate unspecific imager strands; yellow spots indicate the colocalization of origami and imager strands (left). And the fluorescent steps of DNA walker on origami with different walking step sizes (right). (a,b) Reproduced with permission.^[^ ^77^ ^]^ Copyright 2017, American Chemical Society. c) The schematic diagram of DNA walker on the coverslip. d) Row (top) and subdiffraction (bottom) images indicate that DNA walkers travel over 200 nm in 10 min (scale bars: 500 nm). (c,d) Reproduced with permission.^[^ ^122^ ^]^ Copyright 2017, American Association for the Advancement of Science.

Nowadays, in order to visualize the movement of DNA walkers in single‐molecule level, super‐resolution techniques have been used widely.^[^ ^119^, ^120^, ^121^ ^]^ For instance, Wang et al. designed a DNA walker for amplifying fluorescent signals (**Figure** **13**a).^[^ ^77^ ^]^ As illustrated in the scheme, the target served as DNA walker and moved on the DNA origami randomly while consuming the DNA track. Using the ultrasensitive home‐built confocal microscope, the authors could visualize the walking procedure at single molecular level. As shown in Figure 13b, the green spots were used to visualize the location of DNA origami, yellow spots in the image indicated the colocalization of origami and imager strands. Furthermore, the fluorescence transient steps were counted for determining the number of imager strands. As exemplified in Figure 9b, up to five steps (red line) were achieved, which proved the successful walking and imager binding of DNA walkers. In this work, DNA walker was turned into a fluorescence signal amplifier and the signal produced by target could be converted into a large amount of signal molecules.

To further study the kinetic of DNA walking, Choi et al. applied visible/near‐infrared subdiffraction imaging to reveal the mechanism of DNA walking (Figure 13c).^[^ ^122^ ^]^ Due to that the translocation distance of DNA walker was small (<100 nm) and the reaction rate was slow (<0.1 nm s^−1^), the single‐molecule characterization of DNA walking was complicated. Alternatively, Choi and colleagues introduced super‐resolved fluorescence microscopy to image the stochastic behavior of DNA walkers. In this assay, the SWCNT track was functionalized with RNA strands, which was imaged by super‐resolution NIR‐II. Subsequently, the QD‐decorated DNAzyme walker that could cleave RNA fuel strands was imaged (Figure 13d). In the diffraction‐limited raw images (top), the red spots indicated the RNA track images and green spots represented the QD images. The movement of DNA walker was shown in the raw image with 254 nm shift, and the corresponding subdiffraction images (bottom) showed the translocation of DNA walker clearly. These images showed that the process of DNA walker moved over 200 nm along the track in 10 min. Therefore, the ability of this walking system to identify the kinetics in single‐molecule level benefited to study the single biomolecular reaction in biological system.

## 5 Conclusion

In the past few decades, the remarkable development of DNA walkers has attracted more and more researchers to explore this field. Various strategies have been used to develop DNA walkers, and DNA walker‐based biosensors have exhibited excellent performances in detecting different biomarkers, including nucleic acids, proteins, ions, and bacteria. The strengths of DNA walker‐based biosensors are as follows: first, on account of the high programmability and integrability of DNA nanostructures, DNA walker‐based biosensors can be simply and ingeniously designed. Second, with the rapid development of the recognition elements, the constructed DNA walker‐based biosensors are of high universality for detecting many other biomarkers. Finally, amplified signals can be produced at the aid of DNA walkers. Therefore, the detection limit of DNA walker‐based biosensors down to fM level (**Table** **1**).

**Table 1:** Summary of DNA walker‐based biosensors applied in detecting biomarkers

Biomarker		Transduction type	Detection limit	Ref.
Nucleic acids	Zika viruses	Fluorescent	20 pM	[103]
	Ebola viruses	Fluorescent	3.5 pM	[104]
	HIV‐DNA	Electrochemical	4.86 fM	[96]
	HBV‐DNA	Fluorescent	0.2 nM	[123]
	H5N1 DNA	Fluorescent	60 pM	[124]
	ctDNA	Fluorescent	0.22 fM	[49]
	miRNA‐10b	Fluorescent	5 pM	[105]
	miRNA‐21	Fluorescent	6.1 pM	[97]
	miRNA‐155	Colorimetric	16.7 fM	[125]
	let‐7a	Fluorescent	58 fM	[126]
	miRNA‐182‐5p	Electrochemical	31.13 aM	[127]
	miRNA‐182	Electrochemical	1.0 fM	[128]
	miRNA‐141	Electrochemical	3.0 aM	[129]
	miRNA‐892b	Fluorescent	4.0 pM	[130]
Protein	Digoxin	Electrochemical	80 pM	[106]
	Streptavidin	Electrochemical	16 pM	[106]
	PDGF‐BB	Fluorescent	1 pM	[107]
	Thrombin	Fluorescent	3.43 pM	[107]
	Siglec‐5	Electrochemical	8.9 pM	[131]
	CEA	Electrochemical	0.3 pg mL^−1^	[132]
	AFP	Electrochemical	0.5 pg mL^−1^	[132]
	PSA	Photoelectrochemical	1.5 pg mL^−1^	[133]
	CRP	Electrochemical	0.029 pg mL^−1^	[134]
	CtnI	Electrochemiluminescent	0.016 pg mL^−1^	[135]
	T4 PNK activity	Electrochemical	0.001 U mL^−1^	[136]
Ions	Cu^2+^	Electrochemical	0.33 pM	[114]
	Hg^2+^	Electrochemical	0.52 pM	[115]
	Mn^2+^	Electrochemical	0.28 fM	[137]
	Pb^2+^	SERS	3.55 pM	[25]
Bacteria	*Staphylococcus aureus*	Colorimetric	1 CFU mL^−1^	[85]
	*E. coil*	Fluorescent	28.1 CFU mL^−1^	[138]
	*E. coil O157: H7*	Electrochemical	7 CFU mL^−1^	[118]
	*Salmonella typhimurium*	SERS	4 CFU mL^−1^	[117]
	*Vibrio parahaemolyticus*	Electrochemiluminescent	1 CFU mL^−1^	[139]

However, the DNA walker‐based sensing platforms still face several challenges, providing guidance for the future development of DNA walker‐based biosensors. First, the DNA walker‐based sensing system is complicated. In detail, to accomplish a sensing process, at least four components are involved in the system, including DNA walker, DNA track, driving force, recognition elements, and so on. Hence, the distinct pharmacokinetics, biodistributions, and clearance mechanisms of these different components greatly limited the sensing efficiency. Therefore, new DNA walker‐based sensing system with fewer constituent parts is expected to explore. For example, the driving force of DNA walkers can be replaced with endogenous adenosine triphosphate (ATP). Xian et al. designed the DNA walker driven by endogenous ATP for imaging of intracellular miRNA in situ.^[^ ^140^ ^]^ In addition, the sophisticated reaction also blocks the real‐time sensing process. As a result, simplification of the moving process of DNA walkers is the key to accelerate sensing process. Third, a large number DNA walker‐based biosensors have been developed for detecting nucleic acids, proteins, ions, and bacteria. However, because of lacking specific recognition elements, such as antibodies and aptamers, some pathogens cannot be sensed by DNA walker‐based biosensors. Therefore, more recognition elements targeting pathogens should be explored, which needs great effort in biological‐related fields. DNA walkers‐based biosensors worked well in proof‐of‐concept experiments. A number of DNA walkers‐based biosensors have been developed to detect targets in simulated samples, such as diluted fetal bovine serum and serum, indicating great potential in the application in clinics. However, it remains challenging since the clinical applications were partially restricted by the complexity of biological environment. For example, the integrity of DNA walkers may be destroyed by various enzymes, inspiring more robust design of DNA walkers via the tools of synthetic biology in the near future. Recently, researchers raised a novel strategy that endogenous enzymes can be used to drive DNA walkers. For example, Chen et al. developed a DNA walker that all DNA components were anchored on individual gold nanoparticles.^[^ ^141^ ^]^ And two endogenous enzymes were used to actuate walking. In this way, destructive endogenous enzymes were translated to useful driven force. Meanwhile, the moving of DNA walkers was accelerated without requiring external drivers.

In summary, with the great advances in chemical synthesis techniques of DNA nanostructures, the cost of constructing DNA walkers has been greatly reduced. We are looking forward to significant breakthroughs in designing and applying DNA walker‐based biosensors.

## Conflict of Interest

The authors declare no conflict of interest.
